# Food Sources of Energy and Nutrients in Infants, Toddlers, and Young Children from the Mexican National Health and Nutrition Survey 2012

**DOI:** 10.3390/nu9050494

**Published:** 2017-05-13

**Authors:** Liya Denney, Myriam C. Afeiche, Alison L. Eldridge, Salvador Villalpando-Carrión

**Affiliations:** 1 Nestlé Research Center, Lausanne 1000, Switzerland; myriam.afeichezehil@rdls.nestle.com (M.C.A.); alison.eldridge@rdls.nestle.com (A.L.E.); 2 Children’s Hospital of Mexico Federico Gómez, National Institute of Health, Mexico City 06720, Mexico; salvador.villalpando@MX.nestle.com; 3 Nestlé Infant Nutrition, Mexico City 11520, Mexico

**Keywords:** ENSANUT 2012, infants, toddlers, young children, food sources, energy, nutrients

## Abstract

Food sources of nutrients in Mexican children are not well known. To fill the knowledge gap, dietary intake was assessed in 2057 children using a 24-h dietary recall. All reported foods and beverages were assigned to one of 76 food groups. Percent contribution of each food group to nutrient intake was estimated for four age groups: 0–5.9, 6–11.9, 12–23.9, and 24–47.9 months. Breast milk, infant formula, and cow’s milk were the top sources of energy and nutrients, especially in younger groups. Among infants aged 6–11.9 months, the top food sources of energy included soups and stews, cookies, fruit, tortillas, eggs and egg dishes, and traditional beverages. The same foods plus sweetened breads, dried beans, and sandwiches and tortas were consumed as the top sources of energy among toddlers and young children. Milk, soups, and stews were the top contributors for all nutrients and tortillas, eggs, and egg dishes were among the top contributors for iron and zinc. This study showed that low nutrient-dense cookies, sweetened breads, and traditional beverages were among the core foods consumed early in life in Mexico. This compromises the intake of more nutritious foods such as vegetables and fortified cereals and increases the risk of obesity.

## 1. Introduction

Proper nutrition throughout infancy and early childhood is not only vital for optimal growth and development but also helps to lay the foundation for a child’s future health [1,2]. The diet in infancy and early childhood is marked by high nutrient needs [3], a transition from an all-milk diet to family foods in the first year of life [4], and the development of food preferences that may affect long-term food choices later in life [5].

The quality of a child’s nutrition is shaped by decisions made by parents and caregivers as well as the social and economic environment. At present, Mexico is facing malnutrition characterized by stunting and micronutrient deficiencies in young children from low-income families, iron deficiency anemia in young children, widespread obesity in all age groups, and a high prevalence of non-communicable chronic diseases [6,7,8,9,10]. As for young children, recent studies from the 2012 Mexican National Health and Nutrition Survey (Encuesta Nacional de Salud y Nutrición; ENSANUT 2012) have observed shortcomings in infant and child feeding practices [11]. Examples include a low prevalence of breastfeeding, low consumption of iron-rich foods, and high consumption of sugar-sweetened beverages and sweet foods [12,13]. These shortcomings very likely contribute to the imbalanced nutrient intakes reported in recent dietary surveys, including inadequate intakes of iron, calcium, vitamin D, vitamin E, folate, and fiber and excessive intakes of energy, added sugars, saturated fat, and sodium [14,15,16,17,18].

Quantitative assessment of food sources of energy and nutrients can show what foods are important contributors of nutrients in the population’s diet. This knowledge can assist healthcare professionals to form targeted measures to correct shortcomings. Up to now, detailed quantitative analyses on the dietary sources of nutrients in Mexico have been lacking. One recent study conducted in Mexican children under two years of age reported food sources of energy but not nutrients [12]. To fill the knowledge gap, the aim of this study was to describe and identify the principal sources of energy and nutrients in the diets of infants, toddlers, and young children from the ENSANUT 2012.

## 2. Materials and Methods 

### 2.1. Study Population

ENSANUT is a cross-sectional, population-based survey that characterizes the health and nutritional status of the Mexican population [19]. The survey used a multi-stage, stratified, and clustered sampling system drawn to represent all states, four geographic regions, and socioeconomic strata in Mexico. The data were collected from 50,528 Mexican households, with a response rate of 87% [19]. A total of 2057 children from birth to four years of age were used in the current analysis. The data are presented for four age groups: infants 0–5.9 months (*n* = 182), infants 6–11.9 months (*n* = 229), toddlers 12–23.9 months (*n* = 538), and young children 24–47.9 months (*n* = 1108). The survey protocol and data collection instruments were approved by the Ethics Committee of the Mexican National Institute of Public Health (Instituto Nacional de Salud Pública). Written informed consent was obtained from each eligible person 18 years and older or from the parent or caregiver of participants under 18 years. The characteristics of the study population have been described previously [13]. Briefly, the majority of children (70%) lived in urban areas. Of the primary caregivers, most often the mother (85%) had an elementary and/or secondary education; 70% were unemployed and 47% were married.

### 2.2. Dietary Data Collection

One 24-h dietary recall was collected for each child through a face-to-face interview by trained interviewers with the parent or caregiver. The interviewers asked about all foods and beverages and the amount consumed of each item for the previous 24-h period. Custom recipes or standard recipes developed by the National Institute of Public Health were used to estimate the ingredients in mixed food items. The amount of each food item or ingredient consumed was estimated using common household measurement aids (including spoons, cups, slices, handfuls, etc.) and the information was then converted to grams and milliliters depending on the type of food or beverage consumed. To improve dietary recall data, the ENSANUT 2012 implemented an automated five-step multiple-pass method and collected data on both weekdays and weekend days [16].

Quality control of the dietary intake data was conducted in two stages, as reported previously by Lopez-Olmedo and colleagues [16]. Briefly, in the first stage, the foods reported by a participant were reviewed and information including coding, quantity reported, recipe ingredients, and the context in which the meal or feeding episode took place was scrutinized. In the second stage, energy and nutrient intakes were reviewed to identify implausible values. The ratio of daily energy intake to estimated energy requirement was calculated for each person and each day and transformed to a logarithmic scale to remove outliers below −3 SDs and above +3 SDs. Excessive micronutrient intakes were defined as those that exceeded 1.5 times of the 99th percentile of the observed intake distribution of the nutrient in the corresponding sex and age group [16].

Breast milk consumption was estimated based on the child’s age in months and the total amount of other milk (infant formula and cow’s milk) reported over the course of the recall day [20,21]. For exclusively breastfed infants aged birth to 5.9 months, an average intake of 780 mL/day of human milk was assumed; for partially breastfed infants, the amount of human milk was computed as 780 mL/day minus the amount of infant formula/other milks consumed. For infants aged six to 11 months fed human milk as the sole milk source, the amount of human milk was assumed to be 600 mL/day. For partially breastfed infants, the amount of human milk was computed as 600 mL/day minus the amount of infant formula/other milks consumed. For breastfed young children aged 12 to 17 months, the amount of human milk was computed as 89 mL per feeding occasion. For breastfed young children aged 18 to 23 months, the amount of human milk was computed as 59 mL per feeding occasion [20,21].

### 2.3. Analytic Methods

Energy and nutrient intakes were estimated based on the food composition database from the National Institute of Public Health in Mexico (67% foods) [22] and the food composition tables from the United States Department of Agriculture’s Nutrient Database for Dietary Studies (33% of foods). [23]. To calculate added sugars, the intake of each food was linked at the ingredient level (single foods, standardized recipes) or dish level (custom recipes) to the U.S. Department of Agriculture’s National Nutrient Database for Standard Reference [23] and then further linked to the MyPyramid Equivalents Database [24]. Teaspoon equivalents in the Food Patterns Equivalents Database were converted to grams with the use of the ratio 4.2 g/teaspoon. Vitamin A was estimated in retinol activity equivalents using the following formula [25]: Retinol activity = μg retinol + ½ (μg beta-carotene equivalents/6).

To investigate food sources of energy and nutrients, a list of 76 food groups was formed based on previous dietary intake studies in young children in the USA (Table 1) [26,27,28]. Two trained Mexican dietary research specialists and a nutrition scientist at Nestlé adjusted food groups to incorporate local food culture and reflect the relative role of specific types of foods and beverages in the diets of infants, toddlers, and young children living in Mexico [13]. Some common food mixtures were estimated “as consumed”, such as soups, stews, and mixed dishes, and considered a single food item. All foods, beverages, and nutrient supplements were assigned to one of the 76 food groups (Table 1).

### 2.4. Statistical Analysis

Stata (StataCorp. 2015 Stata Statistical Software: Release 14. College Station, TX, USA: StataCorp LP) was used to create data files, assign individual foods and beverages to food groups, and calculate the contribution of each food group to the overall intake of energy and nutrients. The weighted percentage contribution of each food group for all infants, toddlers, and young children was calculated by adding the amount of a given nutrient provided by each food group for all individuals and dividing by the total intake of that nutrient consumed by all individuals from all foods and beverages. All estimates incorporated appropriate sample weights to reflect nationally representative results. Only food groups that contributed over 1% of the nutrient intake are presented in this study. Sources of energy and nutrients were assessed separately and are presented for the four age groups mentioned above.

## 3. Results

Food sources of energy and 14 nutrients in the diets of infants, toddlers, and young children are presented in Table 2 through 16. In each table, the food groups listed present at least 80% of the total energy or nutrient intake.

### 3.1. Energy, Macronutrients, and Fiber

Different types of milk were the top sources of energy across all age groups but the relative contribution reduced markedly with age. Breast milk, infant formula, and cow’s milk were the first, second, and third sources of energy, collectively contributing 89% of total energy among infants 0–5.9 months (Table 2). These milk sources were still the top three sources of energy among infants 6–11.9 months but the total contribution was lower (53%) as more non-milk foods were consumed. Among toddlers 12–23.9 months, cow’s milk was the first source of energy, infant formula was the third, and breast milk dropped to tenth. Among young children 24–47.9 months, cow’s milk was still the first source of energy but other milk sources were no longer on the list (Table 2).

Foods and other beverages consumed as the top 10 sources of energy among infants aged 6–11 months included soups and stews, cookies, yogurt, fruit, tortillas, eggs and egg dishes, and traditional beverages (Table 2). Food diversity increased with age, but these top sources of energy for 6–11.9-month-old remained in the top 10 sources of energy among toddlers and young children, except for yogurt (Table 2). Other foods added to the top 10 sources of energy in the two older groups were sweetened breads, which ranked fifth among toddlers and third among young children, dried beans, and sandwiches and tortas (Table 2). Among the top 10 sources of energy, cookies, sweetened breads, and traditional beverages collectively provided 7%, 14%, and 15% of total energy among 6–11.9-month-old infants, toddlers, and young children, respectively. These foods, together with other foods and beverages high in sugar, including sweetened tea and coffee, fruit-flavored drinks, carbonated sodas, and candy, provided 19% and 22% of daily energy intake among toddlers and young children, respectively.

Most of the top 10 milk and food sources of energy were also the top sources of protein and fat (Table 3 and Table 4) and saturated fat (Supplementary Table S1) in all age groups. Breast milk, infant formula, and cow’s milk were the top three sources of protein and fat among infants of 0–5.9 months and 6–11.9 months; cow’s milk, soups and stews, and eggs and egg dishes were the top three sources of protein and fat among toddlers and young children (Table 3, Table 4 and Supplementary Table S1). Meats ranked fifth as a source of protein among both toddlers and young children. Tortillas ranked seventh and fourth as sources of protein among toddlers and young children, respectively. Overall, food sources of total fat and saturated fat were similar, with a slightly varied ranking (Table 4 and Supplementary Table S1).

The top contributors to energy were also the top sources of carbohydrate (Table 5). Tortillas ranked as the seventh source of carbohydrate among infants 6–11.9 months, then became the second and first source of carbohydrates among toddlers and young children, respectively. For added sugar, cookies, yogurt, fruit-flavored drinks, sweetened breads, and traditional beverages were the top sources across the age groups from 6–11.9 months onwards (Table 6). The contribution of carbonated sodas to added sugar was higher with increasing age, ranking fourteenth among infants 6–11.9 months, seventh among toddlers, and second among young children. Overall, the ranking of added sugar from traditional beverages was higher than carbonated sodas.

Fruit was the highest ranked source of dietary fiber among infants in both age categories. Among toddlers and young children, tortillas contributed the most fiber, followed by fruit (Table 7). From six months onwards, soups and stews were also an important source of fiber. Other top sources of fiber were vegetables, dried beans, pasta mixed dishes, and sweetened breads.

### 3.2. Vitamins

Breast milk, infant formula, cow’s milk, and soups and stews were the main sources of vitamin A (Table 8), vitamin E (Table 9), folate (Table 10), and other B vitamins (Supplementary Tables 2–5), with a slightly varied order. Among toddlers and young children, in addition to different types of milk and soups and stews, eggs and egg dishes, dried beans, tortillas, breakfast cereals, and sweetened breads were also important sources of vitamin A, vitamin E, and B vitamins. Fruit ranked as the first source of vitamin C among toddlers and young children, followed by cow’s milk, soups and stews, infant formula, and traditional beverages (Table 11). For folate, breast milk was the first source among infants; soups and stews were the first and second source among toddlers and young children, respectively, followed by dried beans and eggs and egg dishes (Table 10).

### 3.3. Minerals and Electrolytes

Among infants, in general, breast milk and infant formula were the top two sources of calcium (Table 12), iron (Table 13), zinc (Table 14), and potassium (Table 15). Among toddlers and young children, in addition to cow’s milk, infant formula, yogurt, soups and stews, tortillas, eggs and egg dishes, and dried beans were also important sources of the above minerals.

Except for young children, infant formula ranked as the first source of iron among both infants 0–5.9 months and 6–11.9 months and toddlers, followed by breast milk, soups and stews, cow’s milk, and eggs and egg dishes (Table 13). Tortillas ranked fifth as a source of iron among infant 6–11.9 months and toddlers. Cow’s milk was the number one source of iron among young children, followed by eggs and egg dishes, tortillas, and sweetened breads. In addition to different types of milk, yogurt and soups and stews were the top sources of potassium. Fruit was another major source of potassium across all age groups, with the contribution higher with increasing age (Table 15). Soups and stews were the highest contributors of sodium among all age groups except infants 0–5.9 months, followed by cow’s milk, eggs and egg dishes, and dried beans (Table 16).

## 4. Discussion

The results of this study provide a comprehensive picture of food sources of energy and nutrients and show the shifts with age among Mexican children aged 0–47.9 months. Previous studies on nutrient intake in this population reported inadequate intakes of iron, calcium, vitamin D, vitamin E, folate, and fiber and excessive intakes of energy, added sugars, saturated fat, and sodium [14,15,16,17,18]. Our data have provided important insights on those findings.

### 4.1. Milk Sources

Overall, breast milk, infant formula, and cow’s milk were the top sources of energy, protein, fat, carbohydrates, vitamins (vitamin A, vitamin E, vitamin C, and B vitamins), and minerals (calcium, iron, zinc, and potassium), especially in younger groups. This is similar to what we observed in studies in the USA [26,29] and in China [30]. However, one difference in this study is that cow’s milk was one of the major sources of energy among infants 6–11.9 months. This is a concern because cow’s milk is considered to be an inappropriate milk for children under the age of one year as early feeding of cow’s milk is associated with an increased risk of developing iron-deficiency anemia [31]. The reasons include its low iron content, poor iron availability, and associated occult intestinal blood loss [32]. 

### 4.2. Low Nutrient-Dense Foods and Beverages

As infants and toddlers have a small stomach capacity but high nutrient needs to support their rapid growth, complementary foods should be nutrient-dense, i.e., relatively low in calories and high in vitamins and minerals [3]. However, low nutrient-dense and energy-rich cookies, sweetened breads, and traditional beverages were consumed as core foods in the diet of Mexican children. 

These observations are very much aligned with previous findings that showed a high proportion of energy was provided by caloric beverages [12,33] and that added sugar consumption was high among Mexican children aged 1–4 years [16,18]. Our study provided further details as to what foods and beverages contributed to the high added sugar consumption and the relative role of each food. In addition, we found that consumption of sweetened foods and beverages started as early as the second six months of life and some food items shifted with increasing age. For example, the contribution of carbonated sodas to added sugar doubled in young children compared to toddlers. It is important that energy-rich foods, which provide little nutritional benefit, are limited [34]. Reduced consumption of cookies, sweetened breads, sugar sweetened beverages along with lower sugar content of traditional beverages would markedly decrease the total intake of added sugar in Mexican children.

### 4.3. Food Sources of Iron

Iron-rich foods are lacking in young Mexican children. Iron is of particular importance after six months of age as the infant’s iron stores, which are laid down during gestation, are declining [35]. Thus, complementary foods need to provide iron, either from animal-source foods or from fortification, as recommended in the official Mexican guidelines on nutrition [36] and a recent Mexican complementary feeding consensus paper [37]. Given the detrimental consequences of iron deficiency disorders on cognitive and neurological development [38], a recent position paper on complementary feeding stressed the recommendation of iron-rich food consumption [34]. Previous studies in Mexico have shown that iron intake of infants did not meet recommendations [14], heme-iron intake was low [17], and iron-deficiency anemia was prevalent (23%) [10]. It has already been reported that complementary feeding practices in Mexico lack animal foods [39,40]. In our study, we found that meats provided less than 1% of energy among infants 6–11.9 month olds and were not among the top 10 food sources of energy in toddlers and young children. As a result, meats did not markedly contribute to the intake of iron or vitamins. Also of note, iron-fortified infant cereal made a minimal contribution to nutrient intake in this population. However, it is important to note that Mexican tortilla flour is fortified with iron [41], which might explain why tortillas appears as the third to fifth sources of iron after 12 months of age.

Surprisingly, cow’s milk was found to be a top source of iron (ranked first among young children). This may be caused by two reasons. One is that a proportion of fortified cow’s milk (19.6%) was grouped into cow’s milk in this study and hence increased the iron contribution from the cow’s milk category. The other reason might be that even though cow’s milk is not high in iron, it is frequently consumed, making it a significant iron source.

### 4.4. Role of Local Foods

The ranking of a food as a source of energy or a nutrient reflects not only the concentration of a nutrient in a food but also the frequency of consumption of the food. Soups and stews were found to be top contributors to energy and almost all nutrients after milk including total fat, saturated fat, and sodium. Soups and stews are frequently consumed in this population [12,13]. Since soups and stews in Mexico typically contain meat (usually chicken), vegetables, and tortillas, it is understandable that these food mixtures can provide a wide range of nutrients.

Tortillas were a top source of energy, protein, carbohydrates, a number of B vitamins, calcium, iron, and zinc, and were the number one source of dietary fiber among toddlers and young children. Again, as a staple food in Mexico, high consumption of tortillas makes them a major contributor to macro- and micronutrients. On the contrary, although the contribution of vegetables (consumed as discrete items rather than as food mixtures) to nutrient intake was minimal, indicating low vegetable consumption, vegetables appeared to be in the top three or top five sources of fiber among infants. This is due to the high content of fiber in vegetables, even though they are infrequently consumed. On the other hand, the fact that tortillas were the number one source of fiber among toddlers and young children suggests that good food sources of fiber are really lacking and explains why fiber intake was low in 87% of children aged 1–4 years in Mexico [16]. In addition to the above, we also found that eggs and egg dishes and dried beans (both among the top 10 sources of energy), were top contributors to a number of key nutrients including protein, vitamin A, folate, iron, zinc, potassium, and fiber (dried beans only) in the diet of this population.

### 4.5. Limitations

This study was cross-sectional in design, so it is not possible to evaluate changes in food sources among the same children as they grow. We used a single day 24-h dietary recall, which may not reflect usual intake. The grouping of food items was designed to reflect local food culture and to help us understand the relative role of specific types of foods and beverages, but the choice of food groups could have had an influence on the rankings. If no detailed information was available, standard recipes were used for foods prepared at home, which could have led to either underestimation or overestimation of certain nutrients. Nevertheless, a major strength of this study is the use of small age categories and food groups to describe, in detail, the food sources of energy and nutrients and shifts with age in children aged 0–47.9 months using a nationally representative sample of Mexico.

## 5. Conclusions

This study provides important insights on food sources of energy and nutrients among Mexican children aged 0–47.9 months. The results show that, in addition to milk sources, other types of foods and beverages commonly consumed in Mexico had major contributions to the intakes of energy and nutrients. Foods and beverages high in sugar such as cookies, sweetened breads, and traditional beverages were among the food items commonly consumed from a very young age and contributed increasingly with age to the intake of energy and added sugar. Milk and soups and stews were top contributors to all nutrients. Tortillas and eggs and egg dishes were among the top contributors to iron and zinc. High-fiber foods like vegetables or dried beans were not the top sources of fiber in the diets of children in Mexico. The intake of more nutrient-dense foods such as vegetables, beans, lean meats, and fortified cereals should be encouraged to help address shortfalls in nutrients. Core foods like soups and stews and eggs and egg dishes were the top contributors to sodium, suggesting that they may be suitable targets for sodium reduction. The findings from this study can assist healthcare professionals to develop food-based recommendations to correct the inadequate or excessive intake of certain nutrients in the diets of infants and young children in Mexico.

## Figures and Tables

**Table 1:** Food group classifications *.

***Milk and Milk Products***	***Vegetables***	Pasta mixed dishes
Breast milk	Baby food (vegetables)	***Sweets***
Infant formula (iron fortified powder or fluid)	Dark green/dark green mixtures	Cookies
Cow’s milk (fluid, powdered, flavored, whole or reduced-fat)	White potatoes or starchy/starchy mixtures	Cakes
Cheese	Deep yellow/deep yellow mixtures	Pies & pastries
Yogurt (baby food yogurt or yogurt)	Vegetables (all other/other mixtures)	Sweetened breads (sweet rolls, doughnuts, muffins)
***Meat/Poultry/Fish/Meat Alternates***	100% vegetable juice	Candy
Baby food (meat)	***Fruit***	Mexican desserts
Meats (chicken, turkey or beef)	Baby food (fruit) (apples and apple mixture, bananas and banana mixtures, pear and pear mixtures, other fruit/fruit mixtures)	Ice cream/frozen yogurt/puddings
Fish/shellfish	Canned fruit	Sugars
Game	Fresh or frozen fruit (bananas, berries, nectarines & peaches, pears, melons, guava, other fruit)	Syrups
Hot dogs/cold cuts/bacon/sausage	100% baby fruit juice	Preserves/jelly
Organ meats	100% fruit juice	Fruit-flavored drinks
Pork/ham	***Mixed Dishes***	Carbonated sodas
Dried beans	Beans & rice, chili, bean mixtures with or without meat	Sweetened tea and coffee
Eggs & egg dishes	Beef or pork with vegetables and /or rice/pasta/potatoes	Artificially sweetened powdered beverages
Peanut/nuts/seeds	Chicken or turkey with vegetables and /or rice/pasta/potatoes	Yakult (skimmed milk fermented with *Lactobacillus casei*)
Vegetarian meat substitutes	Fish or shellfish with vegetables and/or rice/pasta/potatoes	Traditional beverages (*atoles*, *licuados* or *aguas frescas*)
***Grains and Grain Products***	Soups & stews	***Other***
Infant cereal	Meat tacos	Butter
Breakfast cereals (plain cereal, cereal flakes, corn flakes or oatmeal)	Vegetable & cheese tacos	Sour cream (whole or reduced fat)
Bread/rolls/biscuits/bagels	Enchiladas	Salty snacks (grain snacks and those made from starchy vegetables)
Tortillas (plain)	Tortillas with fillings/toppings	Condiments, herbs and seasonings
Bars/cereal/granola	Tamales	Salad dressing
Crackers/pretzels/rice cakes	Sandwiches & tortas	Water
Pancakes/waffles/French toast	Wheat-based pasta mixed dishes	Mineral water
Pasta	Pizza	Salsas, *moles*, *adobo*
Rice	Rice-based mixed dishes	Supplements (fortified or vitamins)

* The food classification scheme was adapted from the one developed by for the Feeding Infants and Toddlers Study [26] and modified to include commonly consumed foods among Mexican children. All foods and beverages were classified into these 76 mutually exclusive categories.

**Table 2:** Food sources of energy among Mexican infants, toddlers, and young children aged 0–47.9 months by age group from ENSANUT 2012.

Rank	Age 0–5.9 Months			Age 6–11.9 Months			Age 12–23.9 Months			Age 24–47.9 Months	
	Food Group	% of Total		Food Group	% of Total		Food Group	% of Total		Food Group	% of Total
**1**	Breast milk	52.6		Breast milk	26.7		Cow’s milk	13.2		Cow’s milk	10.7
**2**	Infant formula	34.3		Infant formula	16.5		Soups & stews	7.8		Tortillas (plain)	8.3
**3**	Cow’s milk	1.9		Cow’s milk	10.2		Infant formula	6.9		Sweetened breads	7.0
**4**	Baby food (vegetables)	1.0		Soups & stews	6.5		Tortillas (plain)	5.4		Soups & stews	5.7
**5**	Infant Cereal	1.0		Cookies	4.3		Sweetened breads	4.9		Eggs & egg dishes	5.3
**6**				Yogurt	3.6		Traditional beverages	4.7		Cookies	4.2
**7**				Fresh or frozen fruit	3.2		Eggs & egg dishes	4.3		Dried beans	4.1
**8**				Tortillas (plain)	3.0		Fresh or frozen fruit	4.1		Sandwiches & tortas	4.1
**9**				Eggs & egg dishes	2.6		Cookies	3.9		Traditional beverages	3.3
**10**				Traditional beverages	1.7		Breast milk	3.2		Fresh or frozen fruit	3.2
**11**				Infant cereal	1.7		Yogurt	3.2		Breakfast cereals	2.9
**12**				Dried beans	1.4		Dried beans	2.8		Meats	2.9
**13**				Sweetened breads	1.0		Meats	2.7		Yogurt	2.9
**14**				100% fruit juice	1.0		Breakfast cereals	2.6		Salty snacks	2.8
**15**				Salty snacks	1.0		Sweetened tea and coffee	2.6		Sweetened tea and coffee	2.2
**16**							Salty snacks	2.2		Rice mixed dishes	2.1
**17**							Pasta mixed dishes	1.9		Carbonated sodas	1.7
**18**							Sandwiches & tortas	1.7		Candy	1.4
**19**							Rice mixed dishes	1.5		Vegetable & cheese tacos	1.2
**20**							100% fruit juice	1.5		Tamales	1.2
**21**							Fruit-flavored drinks	1.3		Fruit-flavored drinks	1.2
**22**							Tamales	1.2		Bread/rolls/biscuits/bagels	1.1
**23**										Beef or pork with vegetables and/or rice/pasta/potatoes	1.1
**24**										Pasta mixed dishes	1.1
**25**										100% fruit juice	1.1
**26**										Meat tacos	1.1
**27**										Chicken or turkey with vegetables and/or rice/pasta/potatoes	1.0
**28**										Enchiladas	1.0
**29**										White potatoes	1.0
**All food groups**		**90.8**			**84.4**			**83.6**			**86.9**

**Table 3:** Food sources of protein among Mexican infants, toddlers, and young children aged 0–47.9 months by age group from ENSANUT 2012.

Rank	Age 0–5.9 Months			Age 6–11.9 Months			Age 12–23.9 Months			Age 24–47.9 Months	
	Food Group	% of Total		Food Group	% of Total		Food Group	% of Total		Food Group	% of Total
**1**	Breast milk	48.0		Breast milk	21.5		Cow’s milk	18.2		Cow’s milk	14.8
**2**	Infant formula	40.1		Infant formula	15.9		Soups & stews	9.8		Eggs & egg dishes	10.2
**3**	Cow’s milk	3.3		Cow’s milk	13.2		Eggs & egg dishes	8.7		Soups & stews	7.2
**4**	Yogurt	1.5		Soups & stews	8.9		Infant formula	6.7		Tortillas (plain)	6.4
**5**	Baby food (vegetables)	1.4		Yogurt	5.3		Meats	6.4		Meats	6.0
**6**				Eggs & egg dishes	4.7		Dried beans	4.4		Dried beans	5.8
**7**				Tortillas (plain)	3.0		Tortillas (plain)	4.3		Sweetened breads	5.0
**8**				Dried beans	2.7		Yogurt	3.5		Sandwiches & tortas	4.9
**9**				Cookies	2.7		Sweetened breads	3.4		Yogurt	3.1
**10**				Meats	2.3		Traditional beverages	2.6		Breakfast cereals	2.5
**11**				Traditional beverages	1.9		Breakfast cereals	2.0		Cookies	2.2
**12**				Infant cereal	1.7		Cookies	1.9		Beef or pork with vegetables and/or rice/pasta/potatoes	1.7
**13**				Chicken or turkey with vegetables and/or rice/pasta/potatoes	1.4		Sandwiches & tortas	1.8		Chicken or turkey with vegetables and/or rice/pasta/potatoes	1.6
**14**							Sweetened tea and coffee	1.8		Traditional beverages	1.6
**15**							Breast milk	1.8		Sweetened tea and coffee	1.6
**16**							Chicken or turkey with vegetables and/or rice/pasta/potatoes	1.4		Rice mixed dishes	1.5
**17**							Pasta mixed dishes	1.1		Meat tacos	1.4
**18**							Salty snacks	1.1		Salty snacks	1.4
**19**							Rice mixed dishes	1.1		Fish/shellfish	1.1
**20**							Tamales	1.0		Bread/rolls/biscuits/bagels	1.1
**21**										Vegetable & cheese tacos	1.0
**22**										Tamales	1.0
**23**										Fresh or frozen fruit	1.0
**24**										Enchiladas	1.0
**All food groups**		**94.3**			**85.2**			**83.0**			**85.1**

**Table 4:** Food sources of fat among Mexican infants, toddlers, and young children aged 0–47.9 months by age group from ENSANUT 2012.

Rank	Age 0–5.9 Months			Age 6–11.9 Months			Age 12–23.9 Months			Age 24–47.9 Months	
	Food Group	% of Total		Food Group	% of Total		Food Group	% of Total		Food Group	% of Total
**1**	Breast milk	56.2		Breast milk	33.6		Cow’s milk	16.8		Cow’s milk	14.2
**2**	Infant formula	36.2		Infant formula	20.0		Infant formula	9.1		Eggs & egg dishes	9.8
**3**	Cow’s milk	3.4		Cow’s milk	13.2		Eggs & egg dishes	7.6		Sweetened breads	9.0
**4**				Soups & stews	4.8		Soups & stews	7.1		Meats	5.5
**5**				Cookies	3.7		Sweetened breads	6.1		Soups & stews	5.3
**6**				Eggs & egg dishes	3.5		Breast milk	4.8		Dried beans	5.1
**7**				Yogurt	3.2		Cookies	4.0		Sandwiches & tortas	5.0
**8**				Traditional beverages	1.5		Meats	4.0		Tortillas (plain)	4.2
**9**				Infant cereal	1.5		Dried beans	4.0		Salty snacks	4.1
**10**				Salty snacks	1.3		Salty snacks	3.0		Cookies	3.9
**11**				Dried beans	1.3		Sandwiches & tortas	2.5		Yogurt	2.7
**12**				Sweetened breads	1.0		Tortillas (plain)	2.4		Breakfast cereals	2.1
**13**				Tortillas (plain)	1.0		Traditional beverages	2.3		Beef or pork with vegetables and/or rice/pasta/potatoes	1.8
**14**							Breakfast cereals	2.2		Tamales	1.6
**15**							Pasta mixed dishes	2.0		Rice mixed dishes	1.6
**16**							Tamales	1.7		Chicken or turkey with vegetables and/or rice/pasta/potatoes	1.5
**17**							Yogurt	2.6		Vegetable & cheese tacos	1.4
**18**							Chicken or turkey with vegetables and/or rice/pasta/potatoes	1.2		Meat tacos	1.4
**19**							White potatoes	1.1		Pasta mixed dishes	1.3
**20**										Enchiladas	1.3
**21**										Traditional beverages	1.2
**22**										White potatoes	1.0
**23**										Sweetened tea and coffee	1.0
**24**										Infant formula	1.0
**All food groups**		**95.8**			**89.6**			**84.5**			**87.0**

**Table 5:** Food sources of carbohydrates among Mexican infants, toddlers, and young children aged 0–47.9 months by age group from ENSANUT 2012.

Rank	Age 0–5.9 Months			Age 6–11.9 Months			Age 12–23.9 Months			Age 24–47.9 Months	
	Food Group	% of Total		Food Group	% of Total		Food Group	% of Total		Food Group	% of Total
**1**	Breast milk	50.3		Breast milk	22.5		Cow’s milk	9.9		Tortillas (plain)	11.9
**2**	Infant formula	33.1		Infant formula	13.9		Tortillas (plain)	8.2		Cow’s milk	8.2
**3**	Cow’s milk	1.7		Cow’s milk	7.2		Soups & stews	7.4		Sweetened breads	6.9
**4**	Baby food (vegetables)	1.3		Soups & stews	7.0		Fresh or frozen fruit	7.4		Soups & stews	5.5
**5**	100% fruit juice	1.3		Fresh or frozen fruit	5.5		Traditional beverages	6.4		Cookies	5.1
**6**	Fresh or frozen fruit	1.2		Cookies	5.3		Infant formula	5.9		Traditional beverages	4.6
**7**	Baby food (fruit)	1.1		Tortillas (plain)	4.9		Sweetened breads	4.9		Breakfast cereals	3.6
**8**	Infant cereal	1.1		Yogurt	3.9		Cookies	4.6		Sandwiches & tortas	3.3
**9**	Sweetened tea and coffee	1.0		Traditional beverages	3.0		Sweetened tea and coffee	3.6		Yogurt	3.3
**10**				Infant cereal	1.8		Yogurt	3.6		Fresh or frozen fruit	5.5
**11**				100% fruit juice	1.7		Breakfast cereals	3.0		Dried beans	3.0
**12**				Fruit-flavored drinks	1.6		100% fruit juice	2.6		Sweetened tea and coffee	2.9
**13**				Dried beans	1.5		Fruit-flavored drinks	2.6		Carbonated sodas	2.9
**14**				Infant cereal	1.2		Breast milk	2.6		Salty snacks	2.5
**15**				Rice mixed dishes	1.0		Pasta mixed dishes	2.2		Rice mixed dishes	2.5
**16**				Sweetened tea and coffee	1.0		Rice mixed dishes	2.0		Fruit-flavored drinks	2.3
**17**				Salty snacks	1.0		Dried beans	2.0		Candy	2.0
**18**				Sweetened breads	1.0		Salty snacks	2.0		100% fruit juice	1.8
**19**				Eggs & egg dishes	1.0		Tamales	1.1		Bread/rolls/biscuits/bagels	1.6
**20**				Vegetables	1.0		Sandwiches & tortas	1.1		Vegetable & cheese tacos	1.2
**21**							Candy	1.0		Pasta mixed dishes	1.2
**22**							Carbonated sodas	1.0		White potatoes	1.1
**23**										Tamales	1.1
**All food groups**		**92.1**			**87.0**			**85.1**			**84.0**

**Table 6:** Food sources of added sugar among Mexican infants, toddlers, and young children aged 0–47.9 months by age group from ENSANUT 2012.

Rank	Age 0–5.9 Months			Age 6–11.9 Months			Age 12–23.9 Months			Age 24–47.9 Months	
	Food Group	% of Total		Food Group	% of Total		Food Group	% of Total		Food Group	% of Total
**1**	Yogurt	30.9		Cookies	26.3		Yogurt	14.9		Traditional beverages	12.0
**2**	Sweetened tea and coffee	14.9		Yogurt	21.6		Cookies	11.8		Carbonated sodas	11.5
**3**	Traditional beverages	13.1		Fruit-flavored drinks	7.5		Sweetened breads	10.4		Sweetened breads	10.3
**4**	Bread/rolls/biscuits/bagels	11.6		Sweetened breads	6.4		Traditional beverages	10.3		Yogurt	10.3
**5**	Mexican desserts	6.0		Ice cream/frozen yogurt/puddings	6.3		Fruit-flavored drinks	8.2		Cookies	7.7
**6**	Cookies	5.8		Traditional beverages	6.4		Sweetened tea and coffee	7.6		Cow’s milk	7.5
**7**	Fruit-flavored drinks	4.5		Candy	4.8		Carbonated sodas	5.6		Sweetened tea and coffee	6.8
**8**	Sweet breads	2.6		Bread/rolls/biscuits/bagels	2.3		Breakfast cereals	4.8		Fruit-flavored drinks	6.6
**9**	Carbonated sodas	2.6		Breakfast cereals	2.0		Candy	4.6		Candy	6.3
**10**	Canned fruit	1.8		Mexican desserts	1.9		100% fruit juice	3.6		Breakfast cereals	5.1
**11**	Preserves/jelly	1.8		Salty snacks	1.8		Cow’s milk	3.0		Ice cream/frozen yogurt/puddings	2.4
**12**	Ice cream, frozen yogurt, puddings	1.0		100% fruit juice	1.7		Cakes	2.0		100% fruit juice	1.9
**13**				Sweetened tea and coffee	1.7		Ice cream/frozen yogurt/puddings	1.8		Bread/rolls/biscuits/bagels	1.6
**14**				Carbonated sodas	1.5		Mexican desserts	1.7		Cakes	1.5
**15**				Canned fruit	1.4		Salty snacks	1.6		Yakult	1.5
**16**				Yakult	1.3		Yakult	1.2		Mexican desserts	1.2
**17**				Sugars	1.1		Bread/rolls/biscuits/bagels	1.1		Sandwiches & tortas	1.1
**18**							Artificially sweetened powdered beverage	1.0		Artificially sweetened powdered beverage	1.0
**All food groups**		**96.6**			**96.0**			**95.2**			**95.3**

**Table 7:** Food sources of fiber among Mexican infants, toddlers and young children aged 0–47.9 months by age group from ENSANUT 2012.

Rank	Age 0–5.9 Months			Age 6–11.9 Months			Age 12–23.9 Months			Age 24–47.9 Months	
	Food Group	% of Total		Food Group	% of Total		Food Group	% of Total		Food Group	% of Total
**1**	Baby food (fruit)	22.1		Fresh or frozen fruit	18.8		Tortillas (plain)	17.8		Tortillas (plain)	19.9
**2**	Fresh or frozen fruit	15.0		Soups & stews	18.0		Fresh or frozen fruit	15.4		Fresh or frozen fruit	11.4
**3**	Vegetables	9.7		Tortillas (plain)	14.8		Soups & stews	12.5		Soups & stews	8.6
**4**	Baby food (vegetables)	9.3		Cookies	5.3		Dried beans	5.7		Dried beans	7.0
**5**	Infant formula	8.1		Vegetables	5.0		Pasta mixed dishes	4.5		Sweetened breads	4.7
**6**	Infant cereal	7.8		Dried beans	4.3		Sweetened breads	3.4		Sandwiches & tortas	4.3
**7**	Soups & stews	5.4		Traditional beverages	4.0		Cookies	3.1		Cookies	3.4
**8**	Pasta mixed dishes	4.3		Infant cereal	2.2		Traditional beverages	5.0		Salty snacks	2.9
**9**	Sweetened tea and coffee	3.1		Baby food (fruit)	2.1		Breakfast cereals	2.6		Traditional beverages	2.9
**10**	Tortillas (plain)	2.9		Pasta mixed dishes	2.0		Vegetables	2.4		Breakfast cereals	2.4
**11**	Yakult	1.6		Eggs & egg dishes	1.6		Salty snacks	2.2		Pasta mixed dishes	2.1
**12**	Canned fruit	1.5		White potatoes	1.6		Rice mixed dishes	2.1		Vegetables	1.9
**13**	100% Vegetable juice	1.0		Chicken or turkey with vegetables and/or rice/pasta/potatoes	1.5		Tamales	2.0		Rice mixed dishes	1.8
**14**	Cookies	1.0		Rice mixed dishes	1.4		Sandwiches & tortas	1.6		Meat tacos	1.8
**15**				Fruit-flavored drinks	1.3		100% fruit juice	1.4		Vegetable & cheese tacos	1.8
**16**				Sweetened breads	1.2		Eggs & egg dishes	1.2		Tamales	1.6
**17**				Salty snacks	1.2		Vegetable & cheese tacos	1.2		Enchiladas	1.6
**18**				Infant formula	1.0		Fruit-flavored drinks	1.2		Eggs & egg dishes	1.4
**19**							White potatoes	1.0		Cow’s milk	1.3
**20**										Bread/rolls/biscuits/bagels	1.2
**21**										White potatoes	1.2
**22**										Fruit-flavored drinks	1.0
**All food groups**		**92.8**			**87.3**			**86.3**			**86.2**

**Table 8:** Food sources of vitamin A among Mexican infants, toddlers, and young children aged 0–47.9 months by age group from ENSANUT 2012.

Rank	Age 0–5.9 Months			Age 6–11.9 Months			Age 12–23.9 Months			Age 24–47.9 Months	
	Food Group	% of Total		Food Group	% of Total		Food Group	% of Total		Food Group	% of Total
**1**	Breast milk	55.2		Breast milk	34.2		Cow’s milk	26.7		Cow’s milk	26.3
**2**	Infant formula	35.3		Infant formula	20.3		Infant formula	10.5		Eggs & egg dishes	12.0
**3**	Baby food (vegetables)	3.0		Cow’s milk	15.6		Soups & stews	9.7		Soups & stews	8.4
**4**	Cow’s milk	1.1		Soups & stews	7.0		Eggs & egg dishes	8.7		Breakfast cereals	6.1
**5**	Infant cereal	1.0		Eggs & egg dishes	2.9		Breast milk	6.0		Traditional beverages	4.2
**6**				Infant cereal	1.8		Traditional beverages	4.2		Fresh or frozen fruit	3.5
**7**				Vegetables	1.5		Breakfast cereals	4.0		Dried beans	3.4
**8**				Traditional beverages	1.4		Dried beans	2.7		Sandwiches & tortas	3.3
**9**				Fresh or frozen fruit	1.0		Fresh or frozen fruit	2.7		Sweetened tea and coffee	2.0
**10**				Breakfast cereals	1.0		100% fruit juice	2.0		Rice mixed dishes	1.9
**11**							Vegetables	1.8		Infant formula	1.7
**12**							Rice mixed dishes	1.5		Yogurt	1.6
**13**							Meats	1.4		Vegetables	1.5
**14**							Cookies	1.2		100% fruit juice	1.4
**15**							Sweetened tea and coffee	1.1		Meats	1.2
**16**							Infant cereal	1.1		Cookies	1.2
**17**										Tamales	1.1
**All food groups**		**95.6**			**86.7**			**85.3**			**80.8**

**Table 9:** Food sources of vitamin E among Mexican infants, toddlers, and young children aged 0–47.9 months by age group from ENSANUT 2012.

Rank	Age 0–5.9 Months			Age 6–11.9 Months			Age 12–23.9 Months			Age 24–47.9 Months	
	Food Group	% of Total		Food Group	% of Total		Food Group	% of Total		Food Group	% of Total
**1**	Infant formula	48.0		Infant formula	24.1		Eggs & egg dishes	11.9		Eggs & egg dishes	14.7
**2**	Breast milk	41.9		Breast milk	21.2		Infant formula	11.3		Cow’s milk	7.9
**3**	Pasta mixed dishes	1.1		Soups & stews	8.8		Cow’s milk	10.1		Soups & stews	6.1
**4**	Baby food (vegetables)	1.1		Cow’s milk	6.9		Soups & stews	9.0		Dried beans	5.5
**5**	Vegetables	1.0		Eggs & egg dishes	5.3		Fresh or frozen fruit	5.6		Tortillas (plain)	5.0
**6**				Fresh or frozen fruit	3.6		Pasta mixed dishes	5.0		Salty snacks	5.0
**7**				Cookies	2.7		Dried beans	4.0		Sandwiches & tortas	4.8
**8**				Tortillas (plain)	2.4		Salty snacks	3.9		Breakfast cereals	4.8
**9**				Pasta mixed dishes	1.9		Breakfast cereals	3.3		Fresh or frozen fruit	4.6
**10**				Infant cereal	1.9		Cookies	3.3		Sweetened breads	3.0
**11**				Vegetables	1.5		Tortillas (plain)	3.2		Pasta mixed dishes	2.6
**12**				Breakfast cereals	1.4		Meats	2.2		Cookies	2.3
**13**				Salty snacks	1.3		Sandwiches & tortas	2.1		Meat tacos	2.2
**14**				Dried beans	1.1		Traditional beverages	1.9		White potatoes	2.0
**15**				Traditional beverages	1.1		Sweetened breads	1.8		Rice mixed dishes	1.8
**16**				Baby food (fruit)	1.1		Breast milk	1.8		Beef or pork with vegetables and/or rice/pasta/potatoes	1.7
**17**							White potatoes	1.5		Infant formula	1.7
**18**							Chicken or turkey with vegetables and/or rice/pasta rice/potatoes	1.4		Chicken or turkey with vegetables and/or rice/pasta/potatoes	1.6
**19**							Tamales	1.3		Vegetable & cheese tacos	1.6
**20**							Rice mixed dishes	1.2		Enchiladas	1.6
**21**							100% fruit juice	1.1		Meats	1.5
**22**										Tamales	1.0
**All food groups**		**93.1**			**86.3**			**86.9**			**83.0**

**Table 10:** Food sources of folate among Mexican infants, toddlers, and young children aged 0–47.9 months by age group from ENSANUT 2012.

Rank	Age 0–5.9 Months			Age 6–11.9 Months			Age 12–23.9 Months			Age 24–47.9 Months	
	Food Group	% of Total		Food Group	% of Total		Food Group	% of Total		Food Group	% of Total
**1**	Breast milk	48.1		Breast milk	20.3		Soups & stews	14.4		Sweetened breads	10.9
**2**	Infant formula	39.0		Soups & stews	15.3		Dried beans	8.3		Soups & stews	10.7
**3**	Baby food (vegetables)	2.8		Infant formula	15.2		Fresh or frozen fruit	8.0		Dried beans	10.2
**4**	Vegetables	2.1		Dried beans	6.3		Eggs & egg dishes	7.5		Eggs & egg dishes	8.8
**5**	Soups & stews	1.3		Fresh or frozen fruit	5.6		Sweetened breads	7.0		Breakfast cereals	7.0
**6**	Fresh or frozen fruit	1.0		Vegetables	4.8		Cow’s milk	6.8		Fresh or frozen fruit	6.4
**7**				Cow’s milk	3.8		Infant formula	6.7		Cow’s milk	4.7
**8**				Eggs & egg dishes	3.8		Breakfast cereals	5.6		Cookies	3.7
**9**				Cookies	3.4		Cookies	3.5		Sandwiches & tortas	3.2
**10**				Sweetened breads	1.6		Rice mixed dishes	2.6		Rice mixed dishes	3.2
**11**				Breakfast cereals	1.5		Vegetables	2.1		Tortillas (plain)	3.0
**12**				Rice mixed dishes	1.4		Tortillas (plain)	2.0		Salty snacks	1.6
**13**				Salty snacks	1.3		Breast milk	1.9		Vegetables	1.6
**14**				Infant cereal	1.3		100% fruit juice	1.8		100% fruit juice	1.5
**15**				Yogurt	1.0		Pasta mixed dishes	1.6		Traditional beverages	1.1
**16**							Salty snacks	1.5		Bread/rolls/biscuits/bagels	1.0
**17**							Traditional beverages	2.5		Enchiladas	1.0
**18**							Tamales	1.3		Pasta mixed dishes	1.0
**19**							Sandwiches & tortas	1.1		Infant formula	1.0
**20**										Yogurt	1.0
**All food groups**		**94.3**			**86.6**			**86.2**			**82.6**

**Table 11:** Food sources of vitamin C among Mexican infants, toddlers, and young children aged 0–47.9 months by age group from ENSANUT 2012.

Rank	Age 0–5.9 Months			Age 6–11.9 Months			Age 12–23.9 Months			Age 24–47.9 Months	
	Food Group	% of Total		Food Group	% of Total		Food Group	% of Total		Food Group	% of Total
**1**	Breast milk	50.5		Breast milk	28.7		Fresh or frozen fruit	17.8		Fresh or frozen fruit	15.1
**2**	Infant formula	36.5		Infant formula	16.5		Cow’s milk	10.8		Cow’s milk	11.7
**3**	100% fruit juice	2.3		Soups & stews	8.8		Soups & stews	9.9		Soups & stews	9.2
**4**	Vegetables	2.2		Fresh or frozen fruit	8.5		Infant formula	8.9		Traditional beverages	8.0
**5**	Baby food (vegetables)	1.6		Cow’s milk	7.4		Fruit-flavored drinks	7.1		Fruit-flavored drinks	7.3
**6**	Baby food (fruit)	1.1		Fruit-flavored drinks	3.3		Traditional beverages	6.6		Dried beans	6.2
**7**				Infant cereal	3.3		100% fruit juice	5.8		Breakfast cereals	4.9
**8**				Vegetables	3.2		Dried beans	4.5		100% fruit juice	4.5
**9**				100% fruit juice	2.2		Breast milk	4.3		Rice mixed dishes	2.6
**10**				Dried beans	1.9		Breakfast cereals	2.9		White potatoes	1.8
**11**				Traditional beverages	1.6		Rice mixed dishes	2.2		Eggs & egg dishes	1.8
**12**				White potatoes	1.1		Vegetables	2.0		Sweetened breads	1.6
**13**				Baby food (fruit)	1.0		Eggs & egg dishes	1.5		Cookies	1.5
**14**							Pasta mixed dishes	1.4		Pasta mixed dishes	1.4
**15**							Cookies	1.2		Vegetables	1.4
**16**										Infant formula	1.3
**17**										Vegetable & cheese tacos	1.3
**18**										Beef or pork with vegetables and/or rice/pasta/potatoes	1.2
**19**										Enchiladas	1.1
**20**										Tamales	1.0
**21**										Sandwiches & tortas	1.0
**All food groups**		**94.2**			**87.5**			**86.9**			**85.9**

**Table 12:** Food sources of calcium among Mexican infants, toddlers, and young children aged 0–47.9 months by age group from ENSANUT 2012.

Rank	Age 0–5.9 Months			Age 6–11.9 Months			Age 12–23.9 Months			Age 24–47.9 Months	
	Food Group	% of Total		Food Group	% of Total		Food Group	% of Total		Food Group	% of Total
**1**	Breast milk	47.2		Breast milk	25.8		Cow’s milk	30.1		Cow’s milk	28.3
**2**	Infant formula	40.5		Infant formula	20.1		Yogurt	11.7		Yogurt	9.8
**3**	Yogurt	2.9		Cow’s milk	16.4		Infant formula	10.0		Tortillas (plain)	7.5
**4**	Cow’s milk	2.6		Yogurt	12.2		Traditional beverages	4.9		Traditional beverages	5.6
**5**	Water	1.1		Soups & stews	3.3		Tortillas (plain)	4.3		Sandwiches & tortas	4.8
**6**				Infant cereal	3.0		Soups & stews	3.7		Breakfast cereals	4.5
**7**				Tortillas (plain)	2.2		Breast milk	3.5		Eggs & egg dishes	3.8
**8**				Traditional beverages	2.0		Eggs & egg dishes	3.1		Sweetened tea and coffee	3.3
**9**				Eggs & egg dishes	1.2		Breakfast cereals	3.0		Sweetened breads	3.3
**10**							Sweetened tea and coffee	2.8		Dried beans	3.2
**11**							Dried beans	1.9		Soups & stews	2.1
**12**							Sweetened breads	1.9		Fresh or frozen fruit	1.5
**13**							Sandwiches & tortas	1.9		Infant formula	1.4
**14**							Fresh or frozen fruit	1.7		Salty snacks	1.3
**15**							Infant cereal	1.3		Candy	1.0
**16**							Salty snacks	1.0		Vegetable & cheese tacos	1.0
**17**										Enchiladas	1.0
**All food groups**		**94.3**			**86.2**			**86.8**			**83.4**

**Table 13:** Food sources of iron among Mexican infants, toddlers, and young children aged 0–47.9 months by age group from ENSANUT 2012.

Rank	Age 0–5.9 Months			Age 6–11.9 Months			Age 12–23.9 Months			Age 24–47.9 Months	
	Food Group	% of Total		Food Group	% of Total		Food Group	% of Total		Food Group	% of Total
**1**	Infant formula	47.7		Infant formula	22.7		Infant formula	10.1		Cow’s milk	9.8
**2**	Breast milk	34.1		Soups & stews	11.3		Soups & stews	9.7		Eggs & egg dishes	8.4
**3**	Cow’s milk	2.1		Breast milk	7.1		Cow’s milk	9.1		Tortillas (plain)	8.3
**4**	Soups & stews	1.8		Cow’s milk	6.7		Eggs & egg dishes	7.0		Sweetened breads	7.8
**5**	Water	1.6		Tortillas (plain)	4.9		Tortillas (plain)	5.9		Soups & stews	6.7
**6**	Baby food (vegetables)	1.6		Cookies	4.9		Sweetened breads	5.8		Breakfast cereals	6.6
**7**	Vegetables	1.5		Infant cereal	4.4		Dried beans	5.3		Dried beans	5.9
**8**	Fresh or frozen fruit	1.2		Dried beans	4.3		Breakfast cereals	5.1		Cookies	4.6
**9**	Baby food (fruit)	1.1		Eggs & egg dishes	3.5		Cookies	4.1		Sandwiches & tortas	3.6
**10**				Baby food (fruit)	2.5		Traditional beverages	3.7		Meats	2.9
**11**				Traditional beverages	2.4		Meats	2.7		Traditional beverages	2.7
**12**				Salty snacks	1.9		Fresh or frozen fruit	2.6		Salty snacks	2.4
**13**				Rice mixed dishes	1.9		Sweetened tea and coffee	2.3		Rice mixed dishes	2.1
**14**				Vegetables	1.8		Salty snacks	2.0		Infant formula	1.7
**15**				Sweetened breads	1.6		100% fruit juice	1.7		Sweetened tea and coffee	1.6
**16**				Breakfast cereals	1.5		Tamales	1.7		100% fruit juice	1.2
**17**				Fresh or frozen fruit	1.4		Rice mixed dishes	1.7		Meat tacos	1.1
**18**				100% fruit juice	1.3		Sandwiches & tortas	1.3		Tamales	1.1
**19**				Sweetened tea and coffee	1.3		Pasta mixed dishes	1.3		Enchiladas	1.0
**20**							Infant cereal	1.2		Bread/rolls/biscuits/bagels	1.0
**21**										Fresh or frozen fruit	1.0
**22**										Beef or pork with vegetables and/or rice/pasta/potatoes	1.0
**All food groups**		**92.7**			**87.4**			**84.3**			**82.5**

**Table 14:** Food sources of zinc among Mexican infants, toddlers, and young children aged 0–47.9 months by age group from ENSANUT 2012.

Rank	Age 0–5.9 Months			Age 6–11.9 Months			Age 12–23.9 Months			Age 24–47.9 Months	
	Food Group	% of Total		Food Group	% of Total		Food Group	% of Total		Food Group	% of Total
**1**	Breast milk	44.3		Breast milk	21.1		Cow’s milk	18.9		Cow’s milk	16.0
**2**	Infant formula	43.3		Infant formula	19.6		Infant formula	9.4		Tortillas (plain)	8.8
**3**	Yogurt	2.2		Cow’s milk	12.6		Soups & stews	7.7		Eggs & egg dishes	7.6
**4**	Cow’s milk	1.9		Yogurt	7.0		Eggs & egg dishes	6.2		Soups & stews	5.9
**5**	Baby food (vegetables)	1.3		Soups & stews	6.8		Yogurt	6.1		Dried beans	5.7
**6**				Tortillas (plain)	3.8		Tortillas (plain)	5.8		Yogurt	5.0
**7**				Infant cereal	3.8		Dried beans	4.2		Breakfast cereals	4.8
**8**				Dried beans	2.9		Breakfast cereals	4.0		Sandwiches & tortas	4.6
**9**				Eggs & egg dishes	2.8		Meats	3.8		Meats	4.3
**10**				Traditional beverages	2.2		Traditional beverages	3.0		Sweetened breads	3.7
**11**				Vegetables	1.4		Sweetened breads	2.2		Salty snacks	1.8
**12**				Cookies	1.3		Breast milk	2.0		Cookies	1.7
**13**				Meats	1.1		Cookies	1.7		Beef or pork with vegetables and/or rice/pasta/potatoes	1.7
**14**				Breakfast cereals	1.0		Sandwiches & tortas	1.7		Traditional beverages	1.6
**15**							Pasta mixed dishes	1.4		Rice mixed dishes	1.5
**16**							Salty snacks	1.4		Meat tacos	1.4
**17**							Infant cereal	1.3		Sweetened tea and coffee	1.4
**18**							Sweetened tea and coffee	1.1		Infant formula	1.4
**19**							Rice mixed dishes	1.0		Vegetable & cheese tacos	1.2
**20**							Tamales	1.0		Enchiladas	1.1
**21**										Tamales	1.1
**22**										Chicken or turkey with vegetables and/or rice/pasta	1.0
**All food groups**		**93.0**			**87.4**			**83.9**			**83.3**

**Table 15:** Food sources of potassium among Mexican infants, toddlers, and young children aged 0–47.9 months by age group from ENSANUT 2012.

Rank	Age 0–5.9 Months			Age 6–11.9 Months			Age 12–23.9 Months			Age 24–47.9 Months	
	Food Group	% of Total		Food Group	% of Total		Food Group	% of Total		Food Group	% of Total
**1**	Breast milk	48.4		Breast milk	20.8		Cow’s milk	15.1		Cow’s milk	14.7
**2**	Infant formula	35.0		Infant formula	14.0		Soups & stews	10.9		Soups & stews	8.6
**3**	Baby food (vegetables)	2.2		Cow’s milk	10.9		Fresh or frozen fruit	8.9		Fresh or frozen fruit	7.2
**4**	Cow’s milk	1.9		Soups & stews	9.9		Infant formula	5.8		Dried beans	6.7
**5**	Vegetables	1.6		Fresh or frozen fruit	6.8		Dried beans	4.8		Tortillas (plain)	5.3
**6**	Fresh or frozen fruit	1.5		Eggs & egg dishes	3.6		Sweetened tea and coffee	4.2		Eggs & egg dishes	4.7
**7**	100% fruit juice	1.5		Yogurt	3.4		Traditional beverages	4.2		Sweetened tea and coffee	4.0
**8**	Sweetened tea and coffee	1.2		Dried beans	2.9		Tortillas (plain)	3.5		Yogurt	3.8
**9**	Baby food (fruit)	1.0		100% fruit juice	2.3		Eggs & egg dishes	3.4		Meats	3.1
**10**				Tortillas (plain)	2.2		100% fruit juice	3.4		Sandwiches & tortas	3.0
**11**				Vegetables	2.0		Yogurt	3.3		Traditional beverages	2.5
**12**				Traditional beverages	1.6		Meats	2.7		Breakfast cereals	2.3
**13**				Infant cereal	1.5		Breast milk	2.3		100% fruit juice	2.3
**14**				Sweetened tea and coffee	1.5		Fruit-flavored drinks	2.3		Salty snacks	2.0
**15**				Fruit-flavored drinks	1.3		Vegetables	1.8		Fruit-flavored drinks	1.9
**16**				Chicken or turkey with vegetables and/or rice/pasta/potatoes	1.2		Breakfast cereals	1.7		Sweetened breads	1.9
**17**							Pasta mixed dishes	1.4		Rice mixed dishes	1.6
**18**							Rice mixed dishes	1.4		White potatoes	1.6
**19**							White potatoes	1.2		Vegetables	1.4
**20**							Salty snacks	1.2		Beef or pork with vegetables and/or rice/pasta/potatoes	1.3
**21**							Sandwiches & tortas	1.2		Chicken or turkey with vegetables and/or rice/pasta/potatoes	1.2
**22**							Sweetened breads	1.0		Vegetable & cheese tacos	1.1
**23**										Meat tacos	1.1
**24**										Enchiladas	1.0
**25**										Tamales	1.0
**All food groups**		**94.3**			**85.9**			**85.7**			**85.3**

**Table 16:** Food sources of sodium among Mexican infants, toddlers, and young children aged 0–47.9 months by age group from ENSANUT 2012.

Rank	Age 0–5.9 Months			Age 6–11.9 Months			Age 12–23.9 Months			Age 24–47.9 Months	
	Food Group	% of Total		Food Group	% of Total		Food Group	% of Total		Food Group	% of Total
**1**	Breast milk	44.6		Soups & stews	16.8		Soups & stews	17.6		Soups & stews	12.2
**2**	Infant formula	37.4		Breast milk	15.9		Cow’s milk	9.9		Eggs & egg dishes	10.6
**3**	Soups & stews	3.0		Infant formula	12.6		Eggs & egg dishes	8.4		Dried beans	7.3
**4**	Baby food (vegetables)	2.5		Cow’s milk	8.7		Dried beans	7.2		Cow’s milk	6.9
**5**	Vegetables	1.7		Eggs & egg dishes	6.0		Meats	4.2		Sandwiches & tortas	5.8
**6**	Cow’s milk	2.6		Cookies	5.0		Infant formula	4.1		Meats	5.1
**7**	Water	1.3		Dried beans	3.6		Sweetened breads	3.4		Sweetened breads	4.6
**8**	Infant cereal	1.0		Yogurt	2.3		Cookies	3.2		Salty snacks	3.9
**9**				Salty snacks	1.9		Salty snacks	3.2		Rice mixed dishes	3.3
**10**				Chicken or turkey with vegetables and/or rice/pasta/potatoes	1.9		Traditional beverages	3.2		Tortillas (plain)	3.3
**11**				Infant cereal	1.7		Rice mixed dishes	2.9		Cookies	3.2
**12**				Traditional beverages	1.6		Pasta mixed dishes	2.8		Breakfast cereals	2.5
**13**				Meats	1.4		Breakfast cereals	2.7		Beef or pork with vegetables and/or rice/pasta/potatoes	2.1
**14**				Rice mixed dishes	1.4		Tortillas (plain)	2.1		White potatoes	1.6
**15**				Tortillas (plain)	1.1		Sandwiches & tortas	2.0		Bread/rolls/biscuits/bagels	1.6
**16**				Vegetables	1.1		White potatoes	1.6		Vegetable & cheese tacos	1.5
**17**				Pasta mixed dishes	1.1		Beef or pork with vegetables and/or rice/pasta/potatoes	1.5		Pasta mixed dishes	1.5
**18**							Breast milk	1.2		Tamales	1.4
**19**							Chicken or turkey with vegetables and/or rice/pasta/potatoes	1.1		Enchiladas	1.3
**20**							Sweetened tea and coffee	1.0		Chicken or turkey with vegetables and/or rice/pasta/potatoes	1.3
**21**							Bread/rolls/biscuits/bagels	1.0		Carbonated sodas	1.1
**22**										Meat tacos	1.0
**All food groups**		**94.1**			**84.1**			**84.3**			**83.1**

## Supplementary Materials

The following are available online at http://www.mdpi.com/2072-6643/9/5/494/s1, Table S1: Food sources of saturated fat, Table S2: Food sources of thiamine, Table S3: Food sources of riboflavin, Table S4: Food sources of niacin, Table S5: Food sources of vitamin B6.
