# Supplementary material for: Food Sources of Energy and Nutrients in Infants, Toddlers, and Young Children from the Mexican National Health and Nutrition Survey 2012
Source: Nutrients. 2017 May 13;9(5):494. doi: 10.3390/nu9050494 (PMC5452224; doi:10.3390/nu9050494)
Supplement: Supplementary file 1 [file nutrients-09-00494-s001.pdf]

Table S1. Food sources of saturated fat among Mexican infants, toddlers and young children aged 0-47.9 months by age group from ENSANUT 2012.

	Age 0-5.9 months		Age 6-11.9 months		Age 12-23.9 months		Age 24-47.9 months	
**Rank**	**Food Group**	**% of Total**	**Food Group**	**% of Total**	**Food Group**	**% of Total**	**Food Group**	**% of Total**
**1**	Breast milk	56.6	Breast milk	34.6	Cow's milk	23.3	Cow's milk	20.2
**2**	Infant formula	36.1	Infant formula	21.5	Infant formula	10.3	Sweetened breads	11.2
**3**	Cow's milk	3.4	Cow's milk	14.4	Sweetened breads	7.1	Eggs & egg dishes	7.6
**4**			Yogurts	4.1	Eggs & egg dishes	5.9	Sandwiches & tortas	4.8
**5**			Soups & stews	4.0	Breast milk	5.7	Salty snacks	4.6
**6**			Cookies	2.9	Soups & stews	5.1	Soups & stews	4.0
**7**			Eggs & egg dishes	2.5	Yogurts	4.0	Yogurts	4.0
**8**			Traditional beverages	1.7	Cookies	3.7	Cookies	3.8
**9**			Salty snacks	1.4	Salty snacks	3.2	Meats	3.6
**10**			Infant cereal	1.4	Meats	3.0	Dried beans	3.1
**11**			Sweetened breads	1.1	Traditional beverages	2.8	Breakfast cereals	2.7
**12**					Breakfast cereals	2.5	Tortillas (plain)	2.3
**13**					Sandwiches & tortas	2.3	Tamal	1.7
**14**					Dried beans	1.9	Sweetened tea and coffee	1.6
**15**					Tamal	1.7	Beef or pork with vegetables and/or rice/pasta/potatoes	1.6
**16**					Tortillas (plain)	1.2	Candy	1.4
**17**					Cakes	1.0	Traditional beverages	1.3
**18**					Sweetened tea and coffee	1.0	Infant formula	1.2
**19**							Chicken or turkey with vegetables and/or rice/pasta/potatoes	1.1
**20**							Enchiladas	1.1
**21**							Vegetable & cheese tacos	1.0
**22**							Rice mixed dishes	1.0
**All food groups**		**96.1**		**89.6**		**85.7**		**84.9**

Table S2. Food sources of thiamine among Mexican infants, toddlers and young children aged 0-47.9 months by age group from ENSANUT 2012.

	**Age 0-5.9 months**		**Age 6-11.9 months**		**Age 12-23.9 months**		**Age 24-47.9 months**	
**Rank**	**Food Group**	**% of Total**	**Food Group**	**% of Total**	**Food Group**	**% of Total**	**Food Group**	**% of Total**
**1**	Breast milk	43.8	Infant formula	18.2	Soups & stews	12.7	Cow's milk	11.2
**2**	Infant formula	41.4	Breast milk	17.0	Cow's milk	11.5	Soups & stews	9.8
**3**	Baby food (fruit)	3.0	Soups & stews	13.3	Infant formula	8.3	Sweetened breads	9.1
**4**	Baby food (vegetables)	1.7	Cow's milk	7.2	Sweetened breads	6.5	Tortillas (plain)	7.4
**5**	100% fruit juice	1.5	Dried beans	3.4	Dried beans	5.6	Dried beans	6.7
**6**	Vegetables	1.4	Tortillas (plain)	3.2	Breakfast cereals	5.0	Breakfast cereals	5.8
**7**			Eggs & egg dishes	3.1	Tortillas (plain)	4.9	Sandwiches & tortas	4.2
**8**			Infant cereal	3.1	Fresh or frozen fruits	4.1	Eggs & egg dishes	3.7
**9**			Fresh or frozen fruits	2.8	Traditional beverages	3.8	Fresh or frozen fruits	3.5
**10**			100% fruit juice	2.7	100% fruit juice	3.2	Cookies	2.9
**11**			Cookies	2.6	Cookies	2.8	Rice mixed dishes	2.1
**12**			Traditional beverages	2.4	Eggs & egg dishes	2.7	100% fruit juice	2.0
**13**			Baby food (fruit)	2.3	Pasta mixed dishes	2.2	Bread/rolls/biscuits/bagels	1.6
**14**			Sweetened breads	1.5	Meats	2.0	Meat tacos	1.4
**15**			Vegetables	1.4	Rice mixed dishes	1.8	Meats	1.3
**16**			Breakfast cereals	1.2	Breast milk	1.5	Salty snacks	1.3
**17**			Rice mixed dishes	1.1	Sandwiches & tortas	1.3	Pasta mixed dishes	1.2
**18**					Tamal	1.2	Tamal	1.2
**19**					Vegetables	1.1	White potatoes	1.1
**20**					Salty snacks	1.0	Infant formula	1.1
**21**							Traditional beverages	1.1
**22**							Vegetable & cheese tacos	1.0
**23**							Beef or pork with vegetables and/or rice/pasta/potatoes	1.0
**24**							Sweetened tea and coffee	1.0
**All food groups**		**92.8**		**86.5**		**83.2**		**82.7**

Table S3. Food sources of riboflavin among Mexican infants, toddlers and young children aged 0-47.9 months by age group from ENSANUT 2012.

	**Age 0-5.9 months**		**Age 6-11.9 months**		**Age 12-23.9 months**		**Age 24-47.9 months**	
**Rank**	**Food Group**	**% of Total**	**Food Group**	**% of Total**	**Food Group**	**% of Total**	**Food Group**	**% of Total**
**1**	Breast milk	49.4	Breast milk	24.1	Cow's milk	24.7	Cow's milk	23.9
**2**	Infant formula	39.3	Infant formula	17.6	Eggs & egg dishes	9.9	Eggs & egg dishes	11.4
**3**	Cow's milk	3.3	Cow's milk	16.5	Infant formula	8.6	Breakfast cereals	6.0
**4**	Baby food (vegetables)	1.5	Soups & stews	6.8	Soups & stews	7.4	Soups & stews	5.2
**5**			Eggs & egg dishes	5.1	Breakfast cereals	4.3	Sweetened breads	4.9
**6**			Infant cereal	2.4	Traditional beverages	4.1	Yogurts	3.8
**7**			Fresh or frozen fruits	2.0	Fresh or frozen fruits	3.5	Dried beans	3.7
**8**			Traditional beverages	1.9	Yogurts	3.3	Tortillas (plain)	3.5
**9**			Yogurts	1.7	Sweetened breads	2.8	Sandwiches & tortas	3.5
**10**			Cookies	1.6	Dried beans	2.7	Fresh or frozen fruits	3.0
**11**			Dried beans	1.4	Breast milk	2.4	Sweetened tea and coffee	2.6
**12**			Tortillas (plain)	1.4	Tortillas (plain)	2.2	Traditional beverages	2.6
**13**			Breakfast cereals	1.1	Meats	2.1	Meats	2.6
**14**			Meats	1.0	Cookies	2.0	Cookies	2.2
**15**					Sweetened tea and coffee	1.9	Infant formula	1.2
**16**					Sandwiches & tortas	1.5	Candy	1.0
**17**					Infant cereal	1.3	Rice mixed dishes	1.0
**All food groups**		**93.5**		**84.6**		**84.7**		**81.1**

Table S4. Food sources of niacin among Mexican infants, toddlers and young children aged 0-47.9 months by age group from ENSANUT 2012

	**Age 0-5.9 months**		**Age 6-11.9 months**		**Age 12-23.9 months**		**Age 24-47.9 months**	
**Rank**	**Food Group**	**% of Total**	**Food Group**	**% of Total**	**Food Group**	**% of Total**	**Food Group**	**% of Total**
**1**	Breast milk	45.4	Breast milk	19.3	Soups & stews	14.5	Soups & stews	11.4
**2**	Infant formula	42.1	Infant formula	17.3	Meats	7.8	Tortillas (plain)	8.7
**3**	Baby food (vegetables)	2.2	Soups & stews	15.2	Infant formula	7.3	Sweetened breads	7.9
**4**	Vegetables	1.8	Tortillas (plain)	4.8	Sweetened breads	6.4	Meats	6.8
**5**	Soups & stews	1.5	Cookies	3.3	Tortillas (plain)	5.8	Cow's milk	6.3
**6**	Sweetened tea and coffee	1.2	Infant cereal	3.3	Cow's milk	5.2	Breakfast cereals	5.6
**7**			Fresh or frozen fruits	3.2	Breakfast cereals	4.6	Dried beans	4.5
**8**			Meats	3.1	Dried beans	4.3	Sandwiches & tortas	4.1
**9**			Dried beans	2.5	Sweetened tea and coffee	4.1	Cookies	3.5
**10**			Cow's milk	2.4	Fresh or frozen fruits	3.9	Fresh or frozen fruits	3.4
**11**			Chicken or turkey with vegetables	2.1	Cookies	3.4	Sweetened tea and coffee	3.0
**12**			Eggs & egg dishes	2.0	Rice mixed dishes	2.2	Eggs & egg dishes	2.6
**13**			Vegetables	1.9	Pasta mixed dishes	2.2	Chicken or turkey with vegetables and/or rice/pasta/potatoes	2.3
**14**			Breakfast cereals	1.5	Chicken or turkey with vegetables and/or rice/pasta/potatoes	1.8	Rice mixed dishes	2.2
**15**			Sweetened breads	1.4	Traditional beverages	1.8	Meat tacos	1.7
**16**			Sweetened tea and coffee	1.4	Salty snacks	1.5	Salty snacks	1.7
**17**			Traditional beverages	1.3	Breast milk	1.5	Beef or pork with vegetables and/or rice/pasta/potatoes	1.6
**18**			Rice mixed dishes	1.3	Eggs & egg dishes	1.5	White potatoes	1.3
**19**					Sandwiches & tortas	1.4	Bread/rolls/biscuits/bagels	1.3
**20**					Vegetables	1.2	Vegetable & cheese tacos	1.2
**21**					Tamal	1.2	Fish/shellfish	1.1
**22**					100% fruit juice	1.1	Infant formula	1.0
**23**					White potatoes	1.0	Pasta mixed dishes	1.0
**All food groups**		**94.2**		**87.3**		**85.7**		**84.2**

Table S5. Food sources of vitamin B6 among Mexican infants, toddlers and young children aged 0-47.9 months by age group from ENSANUT 2012

	**Age 0-5.9 months**		**Age 6-11.9 months**		**Age 12-23.9 months**		**Age 24-47.9 months**	
**Rank**	**Food Group**	**% of Total**	**Food Group**	**% of Total**	**Food Group**	**% of Total**	**Food Group**	**% of Total**
**1**	Breast milk	41.7	Breast milk	14.4	Soups & stews	11.0	Soups & stews	8.9
**2**	Infant formula	40.5	Infant formula	13.7	Fresh or frozen fruits	10.0	Tortillas (plain)	8.7
**3**	Baby food (vegetables)	2.7	Soups & stews	11.8	Cow's milk	9.9	Fresh or frozen fruits	7.6
**4**	Fresh or frozen fruits	2.3	Fresh or frozen fruits	8.2	Tortillas (plain)	6.2	Dried beans	7.4
**5**	Vegetables	2.2	Cow's milk	6.5	Dried beans	6.2	Eggs & egg dishes	6.9
**6**	Baby food (fruit)	1.4	Tortillas (plain)	5.8	Meats	5.9	Cow’s milk	6.6
**7**	Soups & stews	1.2	Eggs & egg dishes	4.9	Eggs & egg dishes	5.7	Breakfast cereals	5.9
**8**	Infant cereal	1.0	Dried beans	3.6	Infant formula	5.5	Meats	5.5
**9**			Infant cereal	2.9	Breakfast cereals	4.5	Sandwiches & tortas	3.5
**10**			Vegetables	2.4	Traditional beverages	3.2	Sweetened breads	2.0
**11**			Meats	2.3	Pasta mixed dishes	2.2	Rice mixed dishes	2.0
**12**			100% fruit juice	1.6	100% fruit juice	2.2	White potatoes	1.8
**13**			Chicken or turkey with vegetables and/or rice/pasta/potatoes	1.6	Rice mixed dishes	1.9	Chicken or turkey with vegetables and/or rice/pasta/potatoes	1.8
**14**			Traditional beverages	1.6	Vegetables	1.6	Meat tacos	1.8
**15**			Breakfast cereals	1.4	White potatoes	1.4	Salty snacks	1.7
**16**			White potatoes	1.3	Chicken or turkey with vegetables and/or rice/pasta/potatoes	1.4	Beef or pork with vegetables and/or rice/pasta/potatoes	1.6
**17**			Fruit-flavored drinks	1.0	Salty snacks	1.3	Vegetable & cheese tacos	1.5
**18**			Pasta mixed dishes	1.0	Cookies	1.2	100% fruit juice	1.5
**19**					Tamal	1.2	Cookies	1.4
**20**					Sweetened breads	1.1	Pasta mixed dishes	1.3
**21**					Sandwiches & tortas	1.1	Tamal	1.3
**22**					Breast milk	1.0	Enchiladas	1.2
**23**							Traditional beverages	1.2
**All food groups**		**93.0**		**86.0**		**85.7**		**83.1**
